# Impact of an indigenously produced multi-enzyme complex from *Bacillus subtilis* KT004404 on growth and blood parameters in broiler chicken

**DOI:** 10.1371/journal.pone.0271445

**Published:** 2022-07-27

**Authors:** Aqsa Javaid, Farhan Younas, Ikram Ullah, Masoom Yasinzai

**Affiliations:** Centre for Interdisciplinary Research in Basic Sciences, International Islamic University, Islamabad, Pakistan; Sejong University, REPUBLIC OF KOREA

## Abstract

A 42-days experiment was conducted on a day old birds (n = 400) to evaluate the effect of enzyme supplements in feed on the growth, blood parameters, phosphorous content in bones, and nitrogen retention. Different treatments included: control (C) without enzyme supplement, while the other three groups included enzyme mixture T1 and T2 with two commercially available enzyme mix, and T3 with indigenously produced multi-enzyme complex from *Bacillus subtilis* KT004404. Birds that were fed with indigenously produced multi-enzyme complex showed significant weight gain as compared to other groups. The total feed intake of the birds fed with enzyme supplements was higher than the birds in the control group. The feed conversion ratio was significantly improved (p < 0.05) in treatment groups (T1, T2, T3) as compared to the control. The blood parameters which were analyzed included uric acid, triglycerides, total cholesterol, and serum proteins i.e. globulin and albumin. Birds fed with the enzyme in the group T1, T2 and T3 exhibited higher (p < 0.05) body weight gain. Tibia ash content was significantly higher (p < 0.05) in T1, T2, and T3 as compared to the control. The results of the current study indicate that supplementing poultry feed with the exogenous multi-enzyme produced from *Bacillus subtilis* KT004404 improved the growth of the birds, feed utilization, and exhibited beneficial effects on the blood parameters, phosphorous and nitrogen retention in broiler chicken.

## 1. Introduction

Food security is one of the most recurring challenge in most parts of the world due to which intensive production of meat, eggs, and milk is a necessity, however, this can only be achieved if the farm animals properly consume or digest the nutritious feed [1]. The high cost of conventional feed ingredients such as soybean meal and corn has posed major challenges for the poultry industry in the developing world [2]. Almost 75% of the total cost of animal production is comprised of feed and increasing costs have caused farmers and researchers to look into other less costly unconventional options [3]. Therefore, waste of fruits, vegetables, and other agricultural raw ingredients such as wheat bran and rice straw are also being used as an alternative source of protein, amino acid, vitamins, and energy. Untraditional feed ingredients have also proven to be beneficial in enhancing the growth and performance of poultry birds such as enzyme supplements, vitamins etc. [4].

Poultry performance such as weight and gut health is adversely affected by the inclusion of feed ingredients with anti-nutritional factors such as phytic acid and non-starch polysaccharides (NSP) [5]. To resolve the issue of anti-nutritional impact on metabolizing energy obtained from feed, supplementary enzymes are used that can enhance the nutritional values of the feed ingredients containing phytic acid or NSP [6]. The majority of NSP in poultry is voided through excreta which represents its low digestibility by the animals. NSPs are reported to increase the fluid viscosity due to binding quality with water hence, cause multiple health issues [7]. Similarly, the plant-based feedstuff also contains anti-nutritional phytic acid which contains approximately 29% of the phosphorous but a large amount of phosphorous is unavailable to the birds due to the chelating properties of phytic acid [8]. Other anti-nutritional factors which have been reported to be present in these alternative feed sources include saponins, lectins, and protease inhibitors, which are not digested by the monogastric animals [9]. These anti-nutritional factors decrease the nutrient absorption, increase the digesta viscosity and are also reported to be associated with various pathogenic infections. Addition of exogenous enzymes are known to counter the effects of anti-nutritional factors and improve the economic aspect of poultry production [10].

Few enzymes such as phytase, xylanase, amylase etc. are added as a supplement in the feed as they are not produced in significant amounts in the birds [11]. Diet supplemented with enzymes has proven to be effective in enhancing the growth rate in broilers as the enzymes increase the assimilation and bioavailability of the nutrients and reduce the digestive problems in the animals [12]. Various studies have reported that that the combined application of different enzymes such as xylanase, cellulase, and lipase have synergistic affect which causes enhanced digestibility of the nutrients in the feed [13]. This article aims at analyzing the impacts of multi enzymes complex (phytase, xylanase and amylase) extracted from *Bacillus subtilis* KT004404 as compared to two commercially available enzymes complexes on growth performance, blood parameters, and weight gain in the broiler chicken.

## 2 Materials and methods

### Experimental design

The experiment was performed in the animal facility of International Islamic University, Islamabad, Pakistan. All the protocols were approved by the Institutional Animal Care and Use Committee (IACUC) of the International Islamic University, Islamabad (IIU-IACUC-CIRBS/230-2016) in accordance with the ethical principles and guidelines of the European Union Directive 2007/43 and Regulation 1099/2009. Researchers involved in the handling of birds were trained and certified by the IACUC. Birds were monitored daily for signs such as: diarrhea, rumpled feathers and dehydration. No birds were found dead during the experiments. Birds were euthanized via cervical dislocation during pre-decided time points, and all the mandatory efforts were made to minimize suffering. A total (n = 400) day-old broiler chickens (Cobb-500) were randomly selected for the experiment. The birds were exposed to 17:7 hours of light: dark cycle, feed and water were supplied ad-libitium. Birds were placed in the poultry house with wood shavings covering the floor which were kept dry by routine replacement to fulfill standard hygienic, managerial, and environmental conditions as per Cobb, 2015 guidelines. The birds were vaccinated with Infectious bursal disease vaccine (IBDV) and Newcastle disease vaccine on days 14 and 22 respectively [14].

The birds were nearly equal in body weight and distributed randomly into 4 treatment groups of 100 birds each in 5 replicates (20 birds each replicate). Control group (C) was fed a commercial diet without an enzyme supplement. Other groups received the diets with enzyme supplements of 1000 g on ton diet and termed as T1, T2, T3 treatment group. The enzyme supplements mixed with the diet in T1 and T2 included commercially available enzyme mixture from WOOJIN, Co. Ltd (Phytezyme) and EWHA, Co.(Biomax) respectively. Phytezyme experimental diet contained xylanase (280 U/mL), phytase (130 U/mL), amylase (168 U/mL) and protease (102 U/mL) while Biomax contained phytase (57 U/mL), xylanase (322 U/mL), amylase (89 U/mL) and protease (258 U/mL).

Ltd enzymes preparation respectively while T3 feed was supplemented with an immobilized enzyme complex (IE) produced from indigenous *Bacillus subtilis* KT004404 and control group (C) contained no extracellular enzyme supplement. The study was terminated on the 42nd day. The mortality rate of the birds was recorded regularly during the experimental period. Mash Diet provided to the birds contained similar caloric value and same protein level across all the groups.

### Feed compositional analysis

The chemical composition of the feed was determined through proximate analysis as shown in (Table 1) and ingredients were analyzed according to the proposed methods of (AOAC 2000). Dry Matter was determined by placing the samples in a hot air oven at 650°C for 12 hours (method 930.15). Micro-Kjeldahl method was used for crude protein analysis and the ash content of the feed was analyzed by burning the sample content overnight at 650°C (AOAC 942.05) while ether extract was determined by the Soxhlet method (AOAC 920.39). The enzyme activities in the feed.

**Table 1:** Nutrient composition of diet fed to broilers and proximate analysis of feed ingredients.

Feed Ingredient (g/kg)	Starter	Finisher
Corn	300.02	550.55
Soybean meal (46% CP)	325.23	179.6
Rice grits	200.49	120.67
Sunflower meal	50	42.78
Fish meal	25	40
Limestone	12	12
Canola	73.56	43.2
Dicalcium phosphate	10	7.5
Vitamin premix 1^a^	2.2	2.2
Molasses	1.5	1.5
Composition Analysis		
Metabolic energy	2985 kcal /kg	3122 kcal/ kg
Linoleic acid %	0.14	0.18
Methionine %	0.48	0.44
Lysine %	1.24	1.09
Tryptophan %	0.29	0.20
Crude Fiber %	4.95	6.14
Crude Protein %	23.76	21.88
Crude Fat %	5.86	4.57
Ash content %	7.52	7.98
Moisture %	9.6	10.9

^a^Vitamin premix per kg: retinyl acetate (12,000 UI), cholecalciferol (2500 UI), menadione (2 mg), riboflavin (5 mg), niacin (0.04g), cobalamin (1.5 mcg), folic acid (0.5 mg), biotin (0.05 mg), selenium (0.2 mg).

### Enzyme and treatments

The enzyme activities in the enzyme mixture was determined by using xylan, sodium phytate starch and casein as substrate for xylanase, phytase, amylase and protease assays respectively [15–17] One unit of enzyme activity (xylanase, phytase, amylase, protease) was defined as the amount of enzyme which can liberate 1 μmol of product (xylose, inorganic phosphate, reducing sugar and tyrosine) from catalyzed substrate under assay conditions in one minute. The extracellular enzyme supernatant obtained from the *Bacillus subtilis* KT004404 contained xylanase (113 U/mL), phytase (132 U/mL) and amylase (110 U/mL) and protease (137 U/mL). The thermal stability of adsorbed and free enzymes was studied by carrying out the reaction in the range of incubating temperature. The results showed that the optimal temperature for immobilized enzymes and few enzymes is between 35–40°C. The stability of the enzyme was enhanced after adsorption as immobilized enzymes showed better relative activities 90% at higher temperature (50–80°C). The free enzymes lost the activity after 7 hours under the heat treatment of 80°C while immobilized enzyme on the medium retained 80 percent of their initial activities at the similar temperature. Thermal stability enhancement of the immobilized enzyme was due to the reason that the molecules are stabilized due to hydrogen bonding or hydrophobic interactions between the functional groups of an amino chain of xylanase, amylase and phytase and the hydroxyl group on the adsorbed medium surface [18]. This improved thermal stability of immobilized xylanase benefits the application of adsorbed enzyme at high-temperature industrial and pelleting processes during feed preparation [19]. T1 experimental diet contained xylanase (280 U/mL), phytase (130 U/mL), amylase (168 U/mL) and protease (102 U/mL) while T2 contained phytase (57 U/mL), xylanase (322 U/mL), amylase (89 U/mL) and protease (258 U/mL).

### Sample collection and analysis of growth performance parameters in broiler

Different parameters such as body weight gain [BWG (grams on period) = BW (g) at the end period—BW (g) in first day], feed intake (FI), and feed conversion ratio [FCR (g feed/g gain) = cumulative feed intake (g)/total weight gain (g)] were recorded for 42 days. At the beginning of the experiment, birds were weighed individually. Mean *initial* body *weights* of day-old chicks were almost similar (41.2 ±0.6 g per bird) among all treatment groups. Later in the experiment, at 6 day intervals, randomly selected 5 birds from each treatment group were weighed using a precision weighing balance (Mettler Toledo, USA) for 42 days.

On day 35^th^, 3 birds from each group were weighed and the blood sample was collected in non-heparinized tubes. These samples were centrifuged at 3500 rpm for 15 minutes and serum was collected and stored at -20°C. Serum total protein was determined by the biuret method (Cannon), albumin, uric acid, triglycerides, and cholesterol concentrations were determined by commercially available kits (Labtest Diagnostica, Brazil). Globulin levels were obtained by the difference between serum total protein concentration and serum albumin.

### Analysis of enzyme in intestinal homogenate and pancreas

Three birds from each replicate per treatment were selected for current studies. International standards to conduct the animal research were followed to avoid the pain and suffering of the birds. Bird’s pancreas and intestinal segments were collected at 4°C in Tris-HCL buffer (pH 8). Tissues were homogenized using Dounce homogenizer (Bio Vision, USA), centrifuged at 6000 rpm for 20 minutes at 4°C and the supernatant was stored at -80°C for analysis of enzymes activities i.e. trypsin, amylases, alkaline phosphatase, and lipase. Amylase activity was determined by following an already reported method [20] while trypsin was measured following the method by [21]. Alkaline phosphatase activity was determined by the method proposed by [22] and lipase activity was determined following) [23] method. Enzymatic units were defined as the ability of the enzyme to hydrolyze one micromole substrate in one minute at 25°C.

### Analysis of phosphorous and nitrogen content in excreta

After the 35^th^ day, three birds per replicate were randomly selected for total excreta collection for five consecutive days to determine the content of excreted phosphorous and nitrogen. After collection, the excreta were processed according to the method proposed by [24] and frozen at -20°C. Phosphorous content was determined by the vanadate-molybdate calorimetric method [25] while nitrogen content was calculated using the Kjeldahl method [26].

### Bone mineral content analysis

At the end of the experiment, 3 birds per treatment were selected for collection of the right tibia for bone mineral content quantification. The tibia of the birds was autoclaved for 30 minutes at 121°C and adhered material was cleaned. Endcaps of the tibia were removed, and each tibia was cut into two pieces [27]. Fat extraction from the tibia was done using ice-cold ethyl ether. The tibia was then dried for 24 hours at 100°C and then heated at 650°C for 24 hours. Ashed samples were dissolved in 25% hydrochloric acid by following the method 968.08 (AOAC 1998.) and then total phosphorous content was calculated using the calorimetric method by the molybdate-vendate method [25].

### Statistical analysis

Statistical analysis of data was done using IBM SPSS Statistics 11.0 software. One-way analysis of variance (ANOVA) univariate and repeated measure models indicated the effect of enzymes supplement on broiler performance, blood parameters, phosphorus content in bone, and nitrogen retention. Group comparisons were made by using Tukey post hoc test. Probabilities (*p*) values less than 0.05 (p<0.05) were considered significant.

## Results

### Broiler growth performance

**Fig 1:**
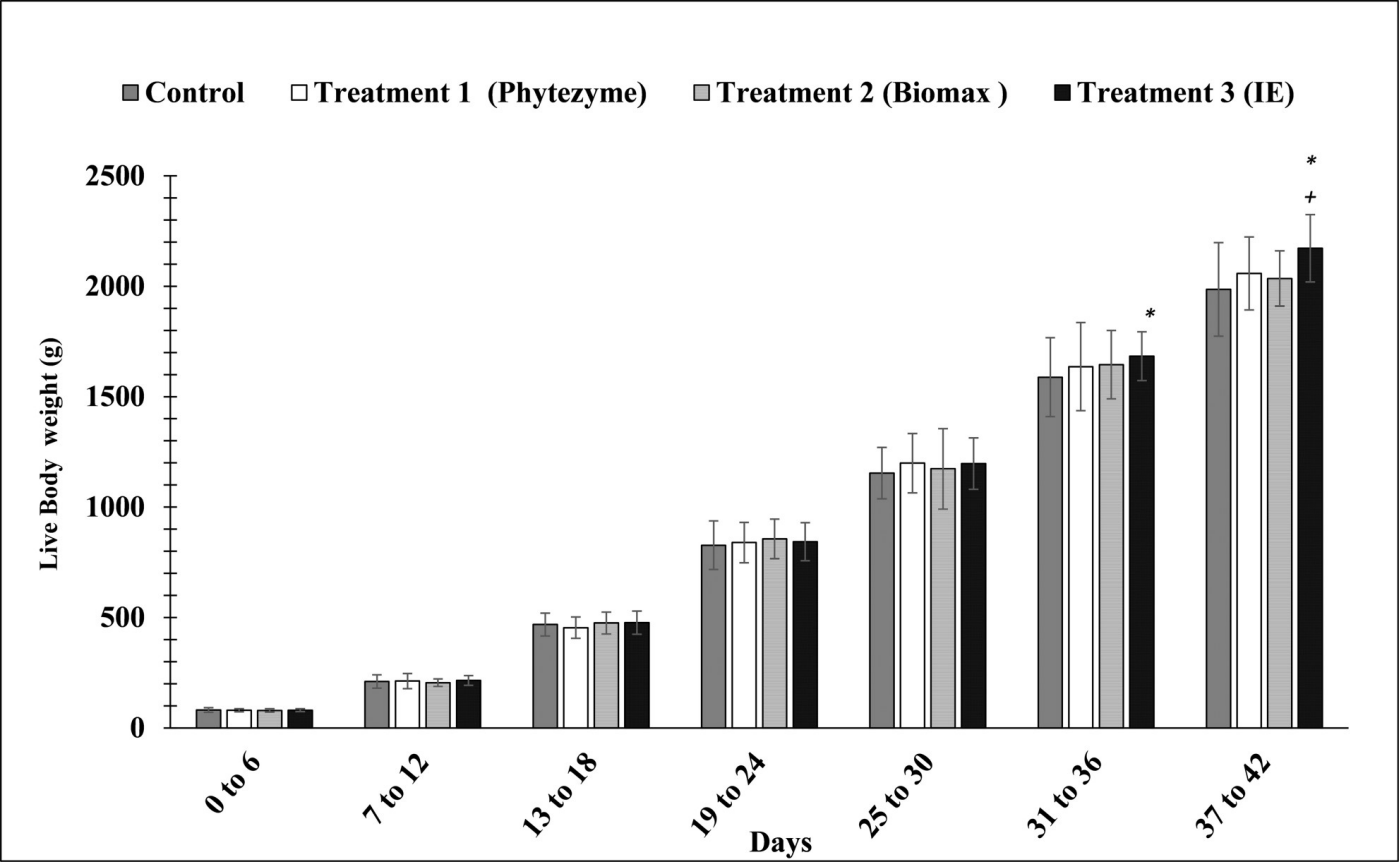
Effects of enzymes’ treatment on body weight gain and growth in birds. Values represent mean ± (SD). Significant differences by Tukey’s test: *p<0.01 from Control group; +p<0.01 from the treatment group (T1 and T2).

The results of the current study revealed that the bodyweight of birds in the experimental group (T3) increased by +2.3% in the birds from 31 to 36 days and +2.8% from 37 to 42 days as compared to groups T1and T2. From 31 to 36 days the average weight gain per bird in T3 group was 486.78 g per bird while from 37 to 42 days the average weight gain was observed to be 488.1 g per bird. This shows that our supplementary enzyme mixture in the feed has a significant impact on increasing the weight in the birds. The effects of various enzyme treatments on body weight between 37 to 42 days in different treatment groups is represented in Fig 1.

**Fig 2:**
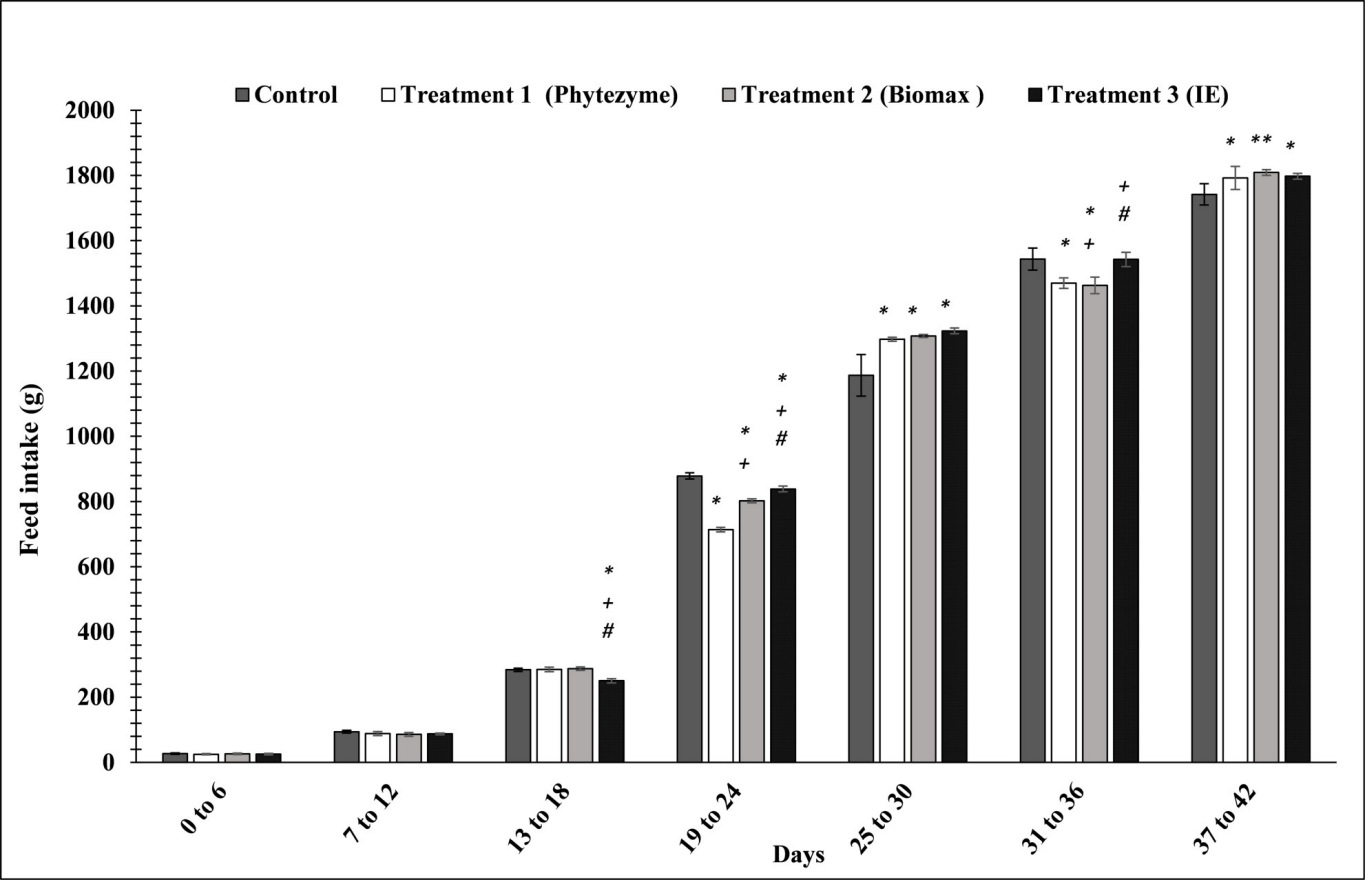
Effects of enzymes’ treatment on feed intake in birds. Values represent mean ± (SD). Significant differences by Tukey’s test: *p<0.01 from control group; ++p<0.01 and +p<0.05 from treatment T1 group and #p<0.01 from T2 group.

The results show that in the birds aged 13 to 18 days, experimental group (T3) feed intake significantly (p<0.05) decreased as compared to control (-11.9%), T1 (-12.26%), and T2 groups (-12.9%). From 19 to 24 days, feed intake in the birds in treatment groups was observed to be significantly reduced by -18% in T1, -8.6% in T2 and -4.4% in T3 as compared to the control. From 25 to 30 days, T1, T2 and T3 groups’ feed intake was significantly higher for T1 (+9.3%), T2 (+10.15%) and T3 (+11.4%) than the control group. However, the feed intake of 31 to 36 days old birds in T1, T2 groups was observed to be significantly reduced as compared to the control group. From 37 to 42 day, birds in the treatment groups showed a significant increase in feed intake (+2.8% for T1, +3.8% for T2, and +3.1% for T3) as compared to the control group (Fig 2).

**Fig 3:**
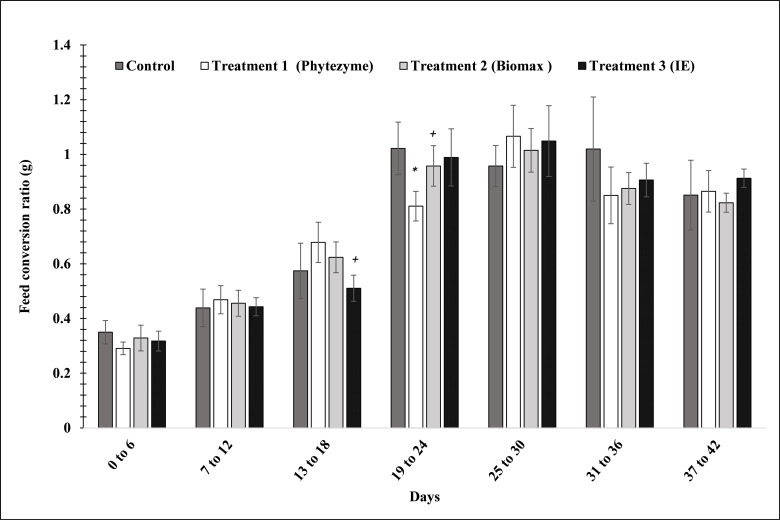
Effects of enzymes’ treatment on FCR in birds. Values represent mean ± (SD). Significant differences by Tukey’s test: *p<0.01 from control; +p<0.05 from T1 group.

When the bird’s FCR was analyzed during the study, results revealed that enzyme treatments have diverse impacts on FCR in the birds depending on the age of the birds. The post hoc test showed a significant (p<0.01) decline in FCR (-23%) of the T3 group as compared to the T1 group from 13 to 18 days, but increase in FCR (+ 20%) in 19 to 24 days old birds as compared to the same group. Whereas, the FCR of T1 from 19 to 24 days was significantly (p<0.05) lower than the control group (Fig 3).

**Fig 4:**
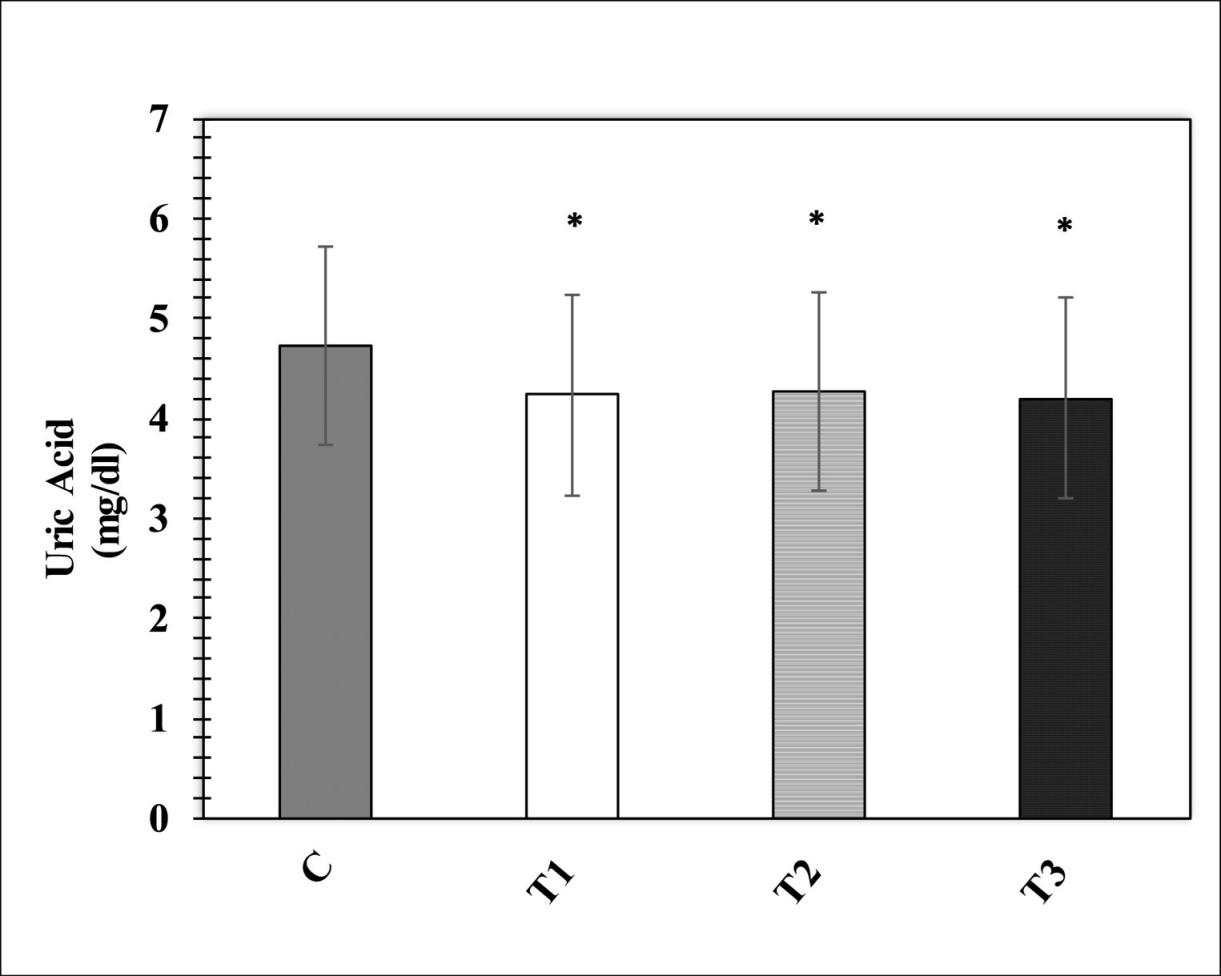
Effects of enzymes’ treatment on serum uric acid. Values represent mean ± (SD). Significant differences by Tukey’s test: *p<0.05 from the control group.

**Fig 5:**
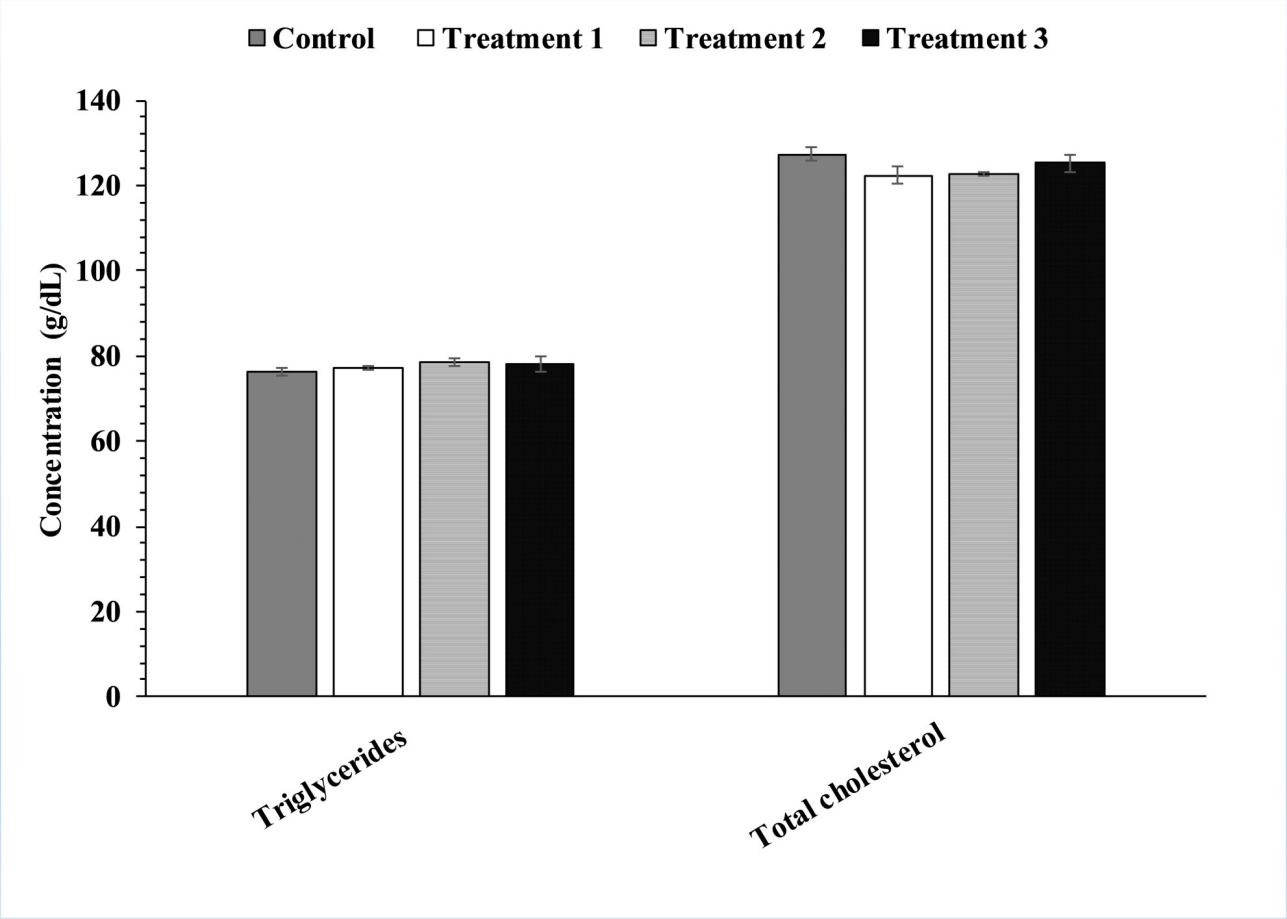
Effects of enzymes treatment on triglycerides and total cholesterol levels. Values represent mean ± (SD).

**Fig 6:**
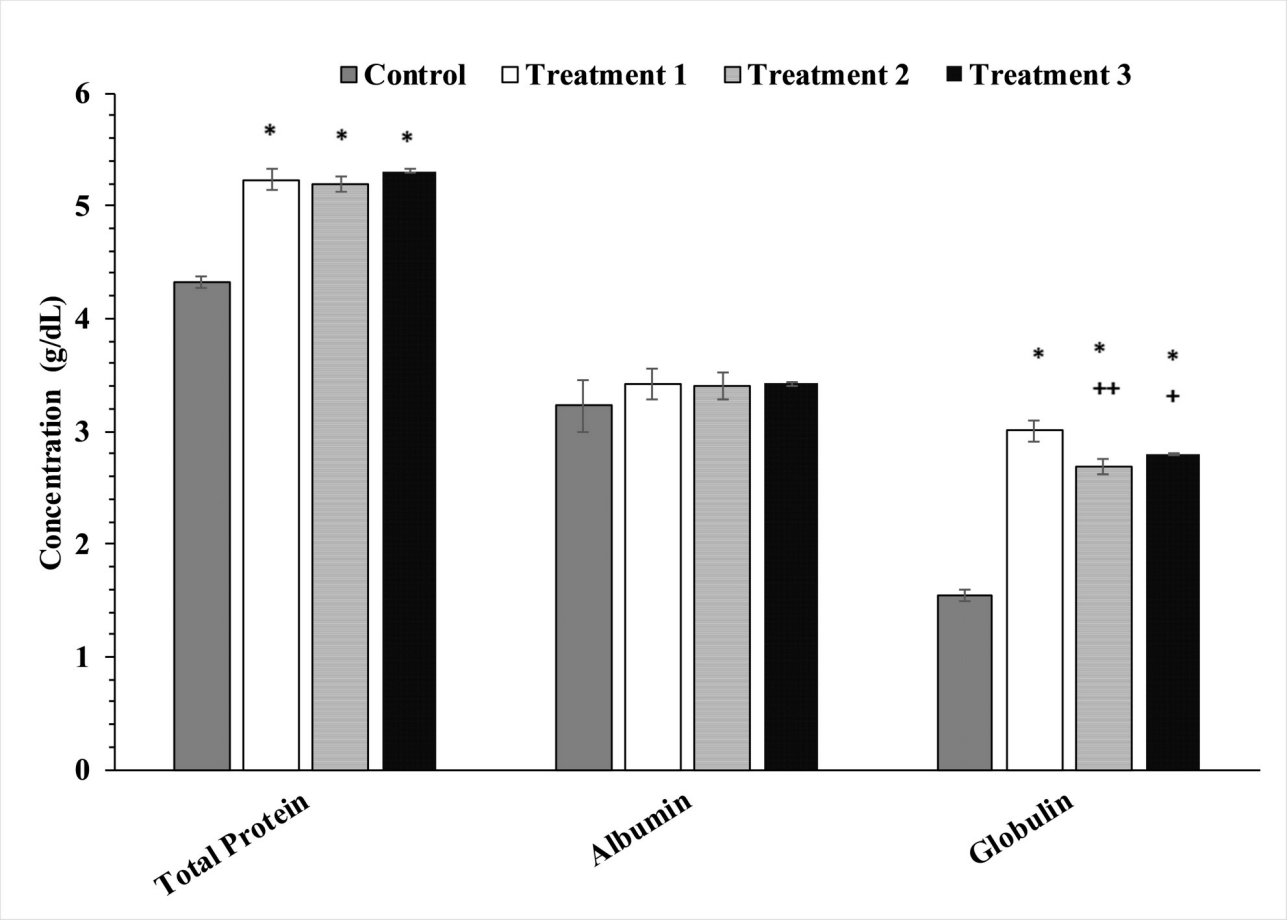
Effects of enzymes treatment on total protein, globulin and albumin levels in blood. Values represent mean ± (SD), *p<0.01 from control group, *p<0.01 from control group, ++p<0.01 and +p<0.05 from T1 group.

When the serum uric acid concentration was analyzed, it was found that that enzymes’ treatment has significantly decreased uric acid concentration in the treatment group T1, T2, T3 as compared to control group (Fig 4). Enzyme’s treatment exhibited no significant effect on triglycerides and total cholesterol levels as shown in Fig 5. When the impacts of mixture of enzyme supplementation on serum protein, globulin and albumin were analyzed it was observed that serum protein (p<0.01) and globulin levels were effected in the treatment groups however, no significant effect was observed on albumin levels. Post-hoc testing for groups’ comparisons and differences revealed that the protein level of T1, T2, and T3 groups were significantly (p<0.01) higher (+ 20% for T1, + 18% for T2 and +23% for T3) than the control group. Globulins level in the treatment groups T1, T2, and T3 were observed to be significantly increased as compared to the control group (Fig 6)

### Enzyme activities in the intestinal homogenate and pancreas

During current studies the impact of enzyme supplements on the protease, α-amylase, trypsin, and lipase activity in the pancreatic and intestinal homogenate was determined. The activity of pancreatic protease in T3 increased (+50%) as compared to control group while trypsin activity declined by -24% for T1, -18% for T2 and -19% for T3 as compared to the control group (Table 2). Similarly, in the intestinal homogenate, the enzyme supplement significantly increased the protease, α-amylase, trypsin, and lipase activity are significantly higher (p<0.05) in the treatment groups as compared to the control group as shown in Table 2.

**Table 2:** Enzyme activities in the pancreases and intestine in the birds.

Enzymatic activities U/mL	C	T1 (Phytezyme)	T2 (Biomax)	T3 (IE)	SEM	p-value
Pancreatic enzymes						
Protease	27.72±4.7	29.6±1.9	36.1±6.4	41.6b^ab^*±4.8	2.0	*
α-amylase	1123.9±74.1	1158±66.4	1058.4±37.7	1115.69±35.0	17.5	0.28
Trypsin	87.2± 5.8	65.9^a^**±1.8	70.9^a^**±2.0	70.6^a^**±0.7	2.5	*
Lipase	65.9±5.6	59.1±3.1	63.6±5.7	69.8±5.9	1.7	0.21
Intestinal Homogenate						
Protease	7.01±0.7	11.61^a^*±0.3	13.51^a^*±1.2	15.77^ab^*±2.3	1.0	*
α-amylase	714.92±64.7	988.39^a^*±25.2	970.66^a^*±54.0	1011.1^a^*±47.4	38.22	*
Trypsin	19.60±2.8	37.66^a^*±1.7	41.99^a^*±0.6	45.62^ab^*±4.1	3.0	**
Lipase	46.23±4.5	47.31±3.1	48.05±2.3	37.82^abc^*±3.3	1.5	*

Values represent mean and standard deviation

Abbreviations: IE, indigenously produced enzyme; SEM, standard error of the mean.

* *p* value ≤ 0.05

** *p* value ≤ 0.01

^a^ Superscript shows significant differences as compared to control (C) of respective enzyme activity (across the row).

^ab^ Superscript shows the significant difference with both C and T1 of respective enzyme activity (across the row).

^abc^ Superscript indicated significant difference as compared to C, T1 and T2 of respective enzyme activity (across the row).

### Phosphorous and nitrogen content in excreta

The effects of various enzymes’ treatment on dry matter intake (DMI), dry matter excreted (DME), nitrogen intake (NI), nitrogen in excreta (NE), phosphorous intake (PI) and phosphorous excreted (PE) is shown in Table 3. Results of the current studies showed that dry matter intake of T1 (609.093 ± 29 g), T2 (611.40 ±36 g) and T3 (621.02 ±19 g) groups were significantly (p<0.05) lower as compared to the control group (C). No significant difference within the treatment groups was observed. The data for DME showed that the treatment groups had a lower level of dry matter in excreta (for T1, T2, T3) as compared to the control. Similarly, the decline in the excreted nitrogen level was only observed in T2 as compared to the control group while phosphorous in excreta was found to be reduced in T1, T2 and T3 by -24%, -21.1% and -11.6% as compared to control. However, enzyme supplements showed no significant effect on the phosphorous intake (PI) between control and treated groups was observed. (Table 3).

**Table 3:** Nitrogen and phosphorous content of broiler excreta.

Parameters	Control	T1 (Phytezyme)	T2 (Biomax)	T3 (IE)	SEM	p- value
DMI (g)	683.81±21.88	609.0933^a^*±29.85	611.40^a^*±36.5	621.02^a^*±19.1	11.4	*
DME (g)	185.27±10.5	153.233^ab^*±6.5	163.44^a^*±4.3	150.05^ab^*±2.3	4.4	**
NI (g)	18.9±0.7	18.7±1.4	17.9±1.2	16.5±1.3	0.41	0.15
NE (g)	10.8±0.4	9.5±0.4	9.2^a^*±0.2	9.3±0.9	0.2	*
PI (g)	4.5±0.6	3.8±0.1	3.7±0.1	3.7±0.2	0.12	0.08
PE (g)	3.17±0.03	2.4^ab^**±0.3	2.5^a^**±0.09	2.8^a^**±0.14	0.09	**

Values represent mean and standard deviation

Abbreviations: DMI, dry matter intake; DME, Dry Matter excreted; NI, Nitrogen intake; NE, nitrogen in excreta; PI, Phosphorous intake; PE, Phosphorous excreted; IE, indigenously produced enzyme; SEM, standard error of the mean

* *p* value ≤ 0.05

** *p* value ≤ 0.01

^a^ Superscript shows significant differences as compared to control (C) across the row.

^ab^ Superscript shows the significant difference with both C and T2 (across the row).

### Bone mineral content

Tibia ash values and phosphorus content in bones for the birds fed with exogenous enzyme supplements in treatment group T1, T2, and T3 were found to be significantly higher (p < 0.05) as compared to control. Exogenous enzyme supplementation was found to have no effect on the length or width of the bones among the treated and control groups as shown in Table 4.

**Table 4:** Determination of tibia ash content in the broiler chicken.

Tibia dimension mm	Control	T1 (Phytezyme)	T2 (Biomax)	T3 (IE)	SEM	*p-*value
Length	75.7± 0.4	75.6± 0.4	75.8± 0.9	75.7± 0.3	0.14	0.95
Width	6.2± 0.3	6.3± 0.3	6.2± 0.26	6.2± 0.05	0.07	0.87
Tibia Ash ^1^ %	37.3^a^ ± 0.3	43.5^b^ ± 0.8	42.9^b^ ± 0.2	42.8^b^± 0.6	0.76	**
Phosphorous ^2^ %	16.2^a^ ± 0.3	17.3^b^ ± 0.3	17.1^b^± 0.3	17.3^b^±0.3	0.16	**

Values represent mean and standard deviation

Abbreviations: IE, indigenously produced enzyme; SEM, standard error of the mean.

** *p* value ≤ 0.01

^a b^ Mean values (C, T1, T2, T3) within a row which do not have common superscript are different (p <0.05).

^1^ Expressed as the percentage of bone ash relative to the weight of bone

^2^ Expressed as a percentage of tibia ash content

## Discussion

The study was performed to evaluate the efficacy of indigenously produced multi-enzyme by *Bacillus subtilis* KT004404 in comparison with the multi-enzyme complexes available in the market. Bodyweight gain (BWG) was not significantly increased up to 30 days as enzyme supplements have no significant impact on the broiler body weight from 0–30 days. However, enzyme supplements containing phytase, xylanase, protease, and amylase were observed to increase the body weight in T3 treatment from 31–42 days. As compared to a commercially available enzymes, the body weight of the birds in the treatment group (T3) significantly increased between 37 to 42 days. Similar results were reported previously in which the food conversion ratio increased due to the addition of exogenous enzymes (cellulase, amylase, and protease) in broiler diet [28]. Endogenous enzyme production is also improved by the addition of exogenous enzymes in diets which increases the nutrients utilization in the feed. Arabinoxylans and phytate are anti-nutritional factors hence, the inclusion of enzyme supplements containing xylanase and phytase improve the broiler performance [29, 30]. An increase in body weight by the addition of enzymes is also in agreement with numerous previously conducted studies [31, 32]. Increase in FCR after the addition of multiple enzymes in the broiler feed indicates improved nutrient utilization [33]. These studies concluded that exogenous enzymes improves the nutrient utilization which is responsible for the increase in the body weight in the groups fed with enzyme supplemented feed as compared to the group fed with a similar level of the nutrient without enzyme supplement [34]. However, the findings are in contradiction with some previous which didn’t report any [35] significant effect of enzyme supplement on the growth performance of broilers. Similarly, no significant impact was observed on the body weight gain and FCR due to the inclusion of exogenous enzymes in diets from 1 to 35 days [36].

In the current studies, broiler feed intake decreased (p < 0.05) with enzyme supplements treatment as compared to the control group (C) from 13 to 24 days. However, in 37 to 42 days old birds the feedd intake (FI) increased significantly in treatments (T1, T2, T3) as compared to the control. The increase in body weight of 37 to 42 days old birds is also an indication of higher feed intake. These results are in agreement with previously conducted studies [2, 31]. Feed intake decreases in the birds fed with exogenous enzymes because the nutrient requirement of birds is fulfilled due to enhanced digestibility in birds [37]. Some earlier studies have reported indigenously produced multi-enzyme to be as effective as a commercially available enzyme, improving the nutrient digestibility, safer to be utilized as a poultry feed supplement and poses no adverse effects on the vital organs in broilers [33–41].

The effect of enzyme supplements on the blood parameters was determined by analyzing uric acid as an indicator of renal function. It was found that uric acid in the serum obtained from treatment T 1, T2, and T3 birds were lower than the group without enzyme supplementation. It suggested that exogenous enzyme preparations increase protein anabolism. In birds, purines are formed from excessive amino nitrogen [42]. The purine bases are degraded into uric acid and excreted out of the body. Increased protein anabolism results in a decline in excessive amino acid hence, lower level of uric acid in the serum. Enzyme inclusion in the feed does not affect serum triglycerides and total cholesterol level[43, 44].

An increase in blood cholesterol and triglycerides by the addition of multi-enzyme in the feed has been reported [30]. Similar results have been reported in numerous previous studies [45, 46] in which increase in blood cholesterol, plasma total protein, and globulin increased by the addition of NSP enzymes in broiler feed. The increase in blood lipid profile after the addition of mixture of enzyme may have association with the improved bile salt functioning and emulsification properties of the chyme that enhances the total cholesterol in the blood [47]. Current studies found higher serum globulins levels in the birds from T1, T2, T3 as compared to the control group which contradicts some earlier reports where no significant effect on the blood serum protein concentration was observed [48–50]. Enhanced concentration of globulin in the serum indicated the enhanced immune response in the birds [51].

The current study found no significant effect on the pancreatic lipase and amylase activity in the groups fed with exogenous enzyme (T1, T2, T3) and the groups without any enzyme supplement (C). However, trypsin activity declined in the treatment groups T1 and T2 while protease script was slightly higher in the group T3. Pancreatic juice contains multiple enzymes such a lipase, trypsin, and amylase are involved in digestive function. Hence, activity of pancreatic enzyme is an effective index to determine the digestive capacity in the birds [52]. The decline in pancreatic trypsin activity by exogenous enzyme supplement is in agreement with some earlier reports [53, 54]. Enzyme supplements impacted intestinal enzyme activity. Current studies showed a decline in lipase activity in the treatment group (T3) fed with indigenous enzyme from *Bacillus subtilis* KT004404 but amylase, trypsin, and protease were found to be higher in the treatment groups (T1, T2, T3) fed with exogenous enzymes. These results are not in agreement with some studies which report decline in amylase and trypsin in the intestinal content [55].

The qualitative and quantitative characteristics of the excreta of broiler showed that phosphate percentage excreted was lower in the groups fed with enzyme supplement in broiler diet as compared to control. This indicates high utilization of minerals and nutrients when the broiler feed was supplemented with exogenous enzyme. Phytase containing exogenous enzymes when added to the poultry diet also reduced in excreted phosphorous [2, 56–58].

The current studies found that Tibia ash percentage in groups fed with exogenous enzyme (T1, T2, T3) was higher than control which indicates improvement in bone mineralization and higher phosphorous deposition in the bone [59]. Percentage of tibia ash was increased when the feed was supplemented with phytase enzyme [58, 60]. The increase in tibia ash and phosphorous content is attributed to higher availability of calcium from the ingredients and organic phosphorous which results in a higher phosphorous deposition in the bones.

## Conclusion

The results of the current experiment demonstrate the efficacy of indigenously produced enzymes from *Bacillus subtilis* KT004404 to be used as feed supplement. The impact of enzyme on the performance of boiler and blood parameters suggests that the indigenous enzyme supplement is effective and safe to be utilized in the poultry feed and has equal efficacy as the commercially available mixture of enzyme supplements. The results provide a significant knowledge base for conducting future studies on the impact of multi-enzyme supplements on the intestinal morphology and microbiome of poultry.
